# Inhibition of Osteoclast Generation: A Novel Function of the Bone Morphogenetic Protein 7/Osteogenic Protein 1

**DOI:** 10.1155/2012/171209

**Published:** 2012-10-24

**Authors:** Thomas Maurer, Gerald Zimmermann, Susanne Maurer, Sabine Stegmaier, Christof Wagner, G. Maria Hänsch

**Affiliations:** Institute for Immunology, University of Heidelberg, Im Neuenheimer Feld 305, 69120 Heidelberg, Germany

## Abstract

Monocytes have the potential to differentiate to either macrophages, dendritic cells, or to osteoclasts. The microenvironment, particularly cytokines, directs the monocyte differentiation. Receptors of NF*κ*B (RANK) ligand, tumor necrosis factor (TNF) *α*, or interleukin- (IL-) 8 have be identified as inducers of osteoclastogenesis, whereas others, such as IL-10 or transforming growth factor (TGF)ß inhibit osteoclast generation or induce differentiation towards a dendritic cell type. We now describe that bone morphogenetic protein (BMP) 7/osteogenic protein- (OP-) 1 inhibited the differentiation of human CD14+ monocytes to osteoclasts. In the presence of BMP7/OP-1 the transcription factors c-Fos and NFATc1, though upregulated and translocated to the nucleus in response to either RANKL or IL-8, did not persist. In parallel, MafB, a transcription factor expressed by monocytes and required for differentiation to macrophages but inhibiting osteoclast generation, was preserved. Because both persistence of NFATc1 and downregulation of MafB are crucial for osteoclastogenesis, we conclude that BMP7/OP-1 inhibits the generation of osteoclasts by interfering with signalling pathways.

## 1. Introduction

Physiological bone turnover, fracture healing, or repair of large bone defects depends on a well-balanced action of bone forming osteoblasts and bone resorbing osteoclasts [1]. A shift towards enhanced osteoclast formation is invariably associated with localized bone loss, as it is seen in patients with periodontitis, osteomyelitis, or implant-associated infections [2–7]. Presumably, the proinflammatory microenvironment created by persistent infections favors the generation of bone resorbing osteoclasts. Indeed, leukocytes recovered from inflamed site expressed increased levels of cytokines or cytokine specific mRNA, respectively [7, 8], which could promote the differentiation of monocytes to osteoclasts. Moreover, in biopsies of patients with osteomyelitis, the density of infiltrating leukocytes correlated with the number of osteoclasts [9].

Differentiation of monocytes to osteoclasts is induced by exogenous stimuli, which activate transcription factors for osteoclast-specific genes. Best studied in the context of osteoclast generation is the receptor activator of Nf*κ*B ligand (RANKL), which binds to its receptor RANK, resulting in translocation and upregulation of NF*κ*B, c-Fos, and NFATc1, transcription factors required for osteoclastogenesis [10–14]. RANKL also induces the transcription of a repressor for MafB [15]. MafB is expressed by myeloid cells, and its downregulation is a prerequisite for differentiation of myeloid cells to osteoclasts [16–18].

We now studied the effect of bone morphogenetic protein (BMP) 7, also known as osteogenic protein (OP)1, on osteoclast generation. BMP7/OP-1 belongs together with other BMPs to the transforming growth factor (TGF) *β* family. BMPs participate in organ development, regeneration, and wound healing, and have numerous, highly diverse biological functions [19, 20]. BMPs bind as dimers to tetrameric receptors which consist of one pair of each, type I receptor and type II receptor subunits [21–23]. These subunits are heterogeneous, and preferential engagement of individual subunits could account for the different biological effects of BMPs, [21–23]. BMPs are especially well studied in bone formation, and they are expressed during fracture healing. Recruitment of stem cells to injured sites and induction of osteoblast proliferation have been described (reviewed in Al-Aql; [1, 24–26])

In recent years, recombinant BMPs, particularly BMP2 and BMP7/OP-1, have been used therapeutically in patients with large bone defects or delayed or impaired fracture healing, with the notion that locally applied BMP would promote bone repair [26–30]. We now tested the effect of BMP7/OP-1 on the differentiation of CD14+ monocytes to osteoclasts, and found that it inhibited osteoclast formation.

## 2. Material and Methods

BMP7/OP-1 was a Gift from Stryker (Kalamazoo, MI, USA).

### 2.1. Culture of Monocytes and Differentiation to Osteoclasts

Monocytes were isolated from the peripheral blood of healthy donors (informed consent was obtained and the institutional guidelines were observed). The blood was layered on Ficoll (Linaris, Wertheim, Germany), and the monocyte fraction was recovered. Monocytes were positively selected using anti-CD14 Micro Beads (Miltenyi Biotec, Bergisch Gladbach, Germany). The CD14+ monocytes were seeded into 24-well dishes (NuncTM, Wiesbaden, Germany) at a concentration of 1 × 10^6^ cells per well and allowed to adhere for 24 hours in medium (RPMI-1640, supplemented with penicillin/streptomycin, all obtained from Gibco/Invitrogen, Karlsruhe, Germany). After 24 h nonadherent cells were removed by washing and the remaining cells were incubated in RPMI supplemented with 10% FCS (PanTM Biotech, Aidenbach, Germany) and M-CSF (25 ng/mL; R&D Systems, Minneapolis, USA). Cells were stimulated with either RANKL (50 ng/mL, PeproTech, Hamburg, Germany) or interleukin- (IL-) 8 (10 ng/mL) or TNF*α* (3 ng/mL) (Calbiochem/Merck, Darmstadt, Germany). OP-1 was used in concentrations of 1 *μ*g/mL. Cultures were incubated at 37°C in 5% CO_2_ for up to 21 days with a change of medium and removal of nonadherent cells after 7 days. For each culture conditions, three parallel wells were prepared. For the followup of the differentiation, the cells were fixed at a given time with 4% PFA for 10 minutes and identified by their morphological appearance (see below).

### 2.2. Characterisation of Osteoclasts

**Figure 1:**
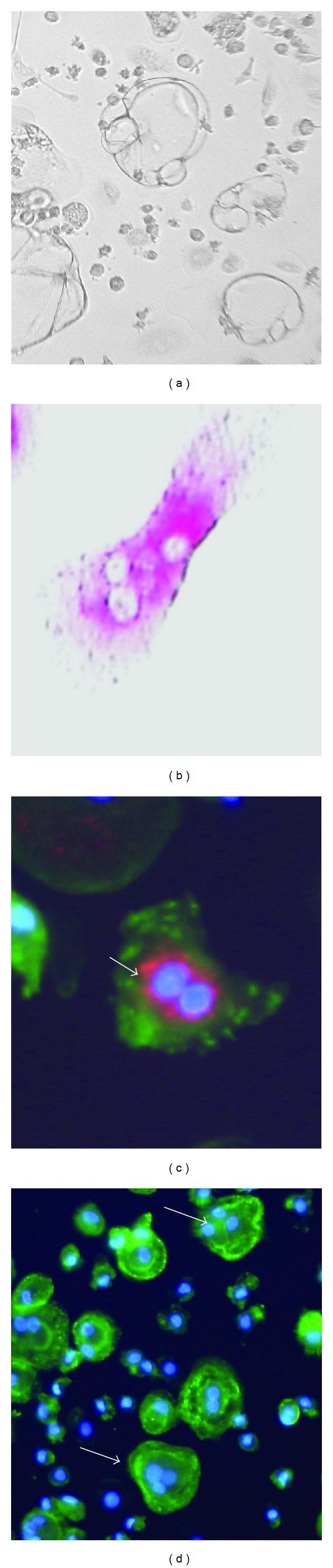
Typical morphological appearance of osteoclasts: after 10 to 14 d in culture (data from day 12 are shown) giant cells were seen ((a); ×400), which expressed TRAP (b), cathepsin K (c) (shown in red, thick arrow), had multiple nuclei (blue) (c), (d), and the typical ring-like distribution of actin (green). Images as shown in (d) were used for quantification.

Osteoclasts were identified by their typical morphology by light microscopy, and by TRAP (tartrate-resistant acid phosphatase) staining, expression of cathepsin K, and actin ring formation. For TRAP staining, cells were fixed with 4% formaldehyde and acetone/alcohol (1 : 1). TRAP staining solution was always freshly prepared and contained 5 mg naphthol AS-MX phosphate (Sigma-Aldrich, Munich, Germany), 0.5 mL N,N dimethylformamide (Schuchardt, Hohenbrunn, Germany) dissolved in a buffer prepared of 30 mg fast red violet LB salt (Sigma-Aldrich), and 0.58 g sodium tartrate (Merck Bioscience, Darmstadt, Germany) in 50 mL 0.1 M sodium acetate pH 5 (Merck Bioscience). Osteoclast formation was evaluated by counting the TRAP+ multinucleated cells. For detection of cathepsin K, the cells were fixed with 4% formaldehyde in PBS. Anti-cathepsin K (Santa Cruz, California, USA), diluted 1 : 50, was added for 60 minutes, followed by washing and incubation with a Cy3 labeled anti-mouse IgG. For actin staining FITC-labeled phalloidin (Sigma-Aldrich) was used in a 1 : 20 dilution for 40 minutes. At last, the nuclei were stained using DAPI (Invitrogen, Oregon, USA), diluted 1 : 30000 for 5 minutes. Cells were mounted with Fluorescent Mounting Medium (Dako, Hamburg, Germany) and fluorescence was visualized using a Digital Fluorescence Microscope (Keyence, Neu-Isenburg, Germany). Osteoclasts were identified by the presence of at least 3 nuclei and an actin ring in typical configuration [31]. Typically, cathepsin K is located at the inside of the actin ring (see Figure 1). For quantification, for each condition 2 to 4 parallel samples were prepared, and cells were counted on images of five high-power fields. Absolute cell numbers for osteoclasts and for nondifferentiated cells were counted and the percentage of osteoclasts in relation to the total cell number was calculated and given as mean of the replicates.

### 2.3. Whole Cell Lysates and Isolation of Nuclei

Cells were lysed with RIPA buffer (tris-buffered saline containing 1% Nonidet P-40, 0.5% sodium deoxycholate, 0.1% sodium dodecyl sulfate, 0.0004% sodium azide, 0.2 M orthovanadate, and 0.5 M phenylmethyl-sulfonyl fluoride) and stored at −80°C until use. For nuclear protein extraction 3 × 10^6^ cells were used and the Nuclear Extraction Kit (Active Motif, Carlsbad, CA) according to the protocol provided by the manufacturer.

### 2.4. ELISA of NF*κ*B and NFATc1

NF*κ*B and NFAT were determined using the TransAM NF*κ*B and TransAM NFATc1 transcription factor assay kit (Active Motif, Carlsbad, CA). The assay is based on a 96-well format, each well containing immobilized oligonucleotides representing the NF*κ*B or NFATc1 consensus sequences, respectively.

### 2.5. Quantitative RT-PCR for IL-8

Fom 10^6^ monocytes mRNA was isolated using the MagnaPure mRNA Isolation Kit I (RAS, Mannheim, Germany) and reversely transcribed using AMV-RT and oligo-(dT) as primer (First Strand cDNA synthesis kit, RAS) according to the manufacturer's protocol. The primer sets specific for IL-1 and cathepsin K, respectively, were optimized for the LightCycler and RT-PCR was performed by SEARCH-LC GmbH, Heidelberg (www.search-lc.com). The copy number of the respective RNA was calculated from a standard curve, obtained by plotting known input concentrations of four different plasmids at log dilutions to the PCR-cycle number (CP) at which the detected fluorescence intensity reached a fixed value. To correct for differences in the content of mRNA, the calculated copy numbers were normalized according to the average expression of two housekeeping genes, cyclophilin B, and *β*-actin. Values were thus given as input adjusted copy number per *μ*L of cDNA and represent the mean of duplicates. 

### 2.6. SDS-PAGE and Western Blot

Samples were boiled 10 min at 95°C and separated by SDS-polyacrylamide gel (12%) electrophoresis, followed by Western-blotting using a Nitrocellulose Transfer Membrane (Whatman, Dassel, Germany). The membrane was probed with the following antibodies: anti-NF*κ*B (Cell Signaling, Danvers, USA), anti-NFATc1 (Santa Cruz Biotechnology inc., Heidelberg, Deutschland), and anti-c-Fos (Cell Signaling), anti-MafB (GenWay, San Diego). The antibodies were diluted in 5% BSA (Sigma-Aldrich), 1 x TBS and 0.1% Tween (Calbiochem/Merck, Darmstadt, Germany) at 4°C over night. The secondary antibodies, peroxidase-labelel anti-mouse IgG, or anti-rabbit IgG (Jackson Immuno Research, Pennsylvania, USA) was added for 30 minutes at room temperature. For detection, Amersham ECL plus Western Blotting Detection System (GE Healthcare Limited, Munich, Germany) was used. As loading controls *β*-actin or the nuclear antigen p84 were used. All experiments were repeated with cells of different donors, and one of at least three experiments is shown as example.

### 2.7. Statistical Analysis

Because of the wide variation among individual donors, all experiments were performed with cells of three to five individuals, and results are expressed as n-fold increase over unstimulated cells. If not stated differently, the data are given as mean ± SD and differences between mean values were calculated by ANOVA or *t*-test for paired samples.

## 3. Results

### 3.1. BMP7/OP-1 Inhibits the Osteoclast Formation

**Figure 2:**
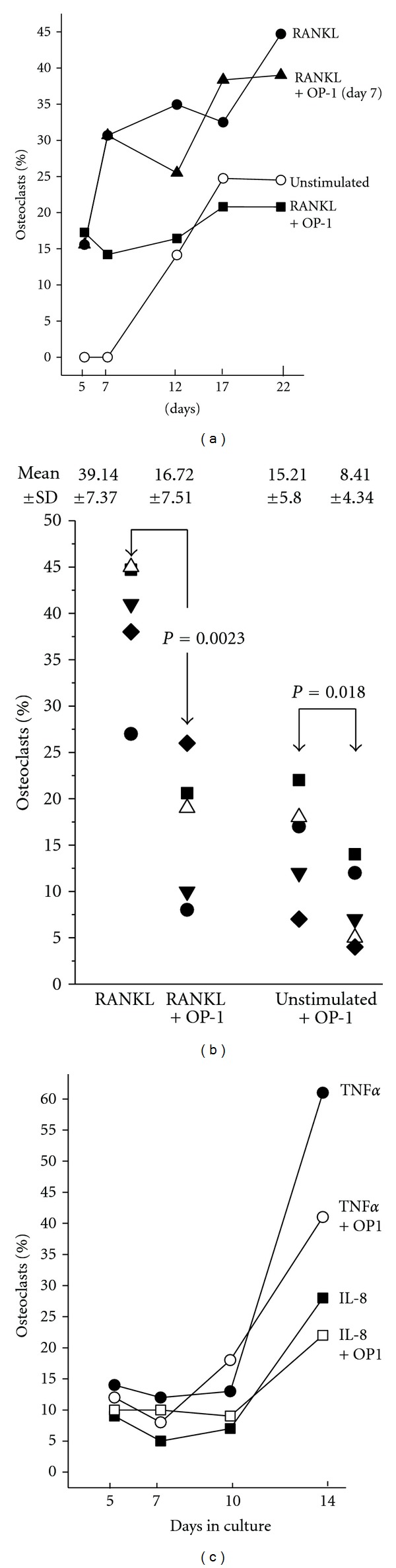
Differentiation of CD14+ monocytes to osteoclasts in culture. (a) Monocytes were cultivated with RANKL (50 ng/mL) (●), RANKL, and OP-1 (1 *μ*g/mL) (■) or cultivated without stimulation (∘). In parallel, cells were cultivated for 7 days with RANKL, and then OP-1 was added (▲). At days 5, 7, 12, 17, and 22 the number of osteoclasts in relation to the total cell number was calculated (given are the mean values of triplicates with cells from one donor). (b) Monocytes of five individuals (each symbol stands for one individual) were cultivated with RANKL or with RANKL+OP-1 or left unstimulated with or without OP-1. After 22 days the percentage of osteoclasts was calculated (the mean values of the groups ± SD are given; the difference between the groups was calculated using *t*-test for paired samples). (c) Monocytes were cultivated with either IL-8 (10 ng/mL; squares) or TNF*α* (3 ng/mL) with (open symbols) or without (closed symbols) OP-1 and the percentage of osteoclasts was calculated at various times in culture (shown is the mean of duplicates and data of two individuals are shown).

CD14+ monocytes isolated from the peripheral blood of healthy donors were cultivated with M-CSF and RANKL. Within 4 to 6 days fusion of monocyte was observed, by days 10 to 14, cells with characteristic osteoclast morphology were seen (multiple nuclei; actin in a ring-like configuration; expression of TRAP and cathepsin K; examples in Figure 1). On average, following activation by RANKL 39.1 ± 7.4% of cells were identified as osteoclasts (mean ± SD of 5 experiments with cells of 5 individual donors, without stimulation 15.2 ± 5.85). When BMP7/OP-1 was added to the cultures, differentiation of monocytes to osteoclasts was inhibited. In a concentration of 1 *μ*g/mL, OP-1 reduced the number of osteoclasts by 57.1 ± 8.2% (determined at day 14 following stimulation with RANKL; mean ± SD of 5 experiments with cells of different donors; the inhibition was statistically significant according to *t*-test for paired samples, *P* = 0.0023) (Figures 2(a) and 2(b)). Increasing the OP-1 dose for up to 5 *μ*g/mL did not enhance its inhibitory activity; with 0.2 *μ*g/mL inhibition was on average 20.0 ± 7.9% and only marginally significant (*P* = 0.038). OP-1 alone did not induce morphological changes of the monocytes, nor did it affect their viability (data not shown). Of note, when OP-1 was added to cells 24 h after stimulation with RANKL the generation of osteoclasts was still inhibited; when it was added after 7 days, the osteoclastogenesis was not affected (Figure 2(a)). An inhibition by OP-1 was also seen for the spontaneous differentiation of osteoclasts (Figure 2(b)) or when osteoclast generation was induced by TNF*α* or by IL-8 (examples in Figure 2(c)). 

### 3.2. Effect of OP-1 on NF*κ*B Translocation, c-FOS Generation, and NFATc1 Translocation

**Figure 3:**
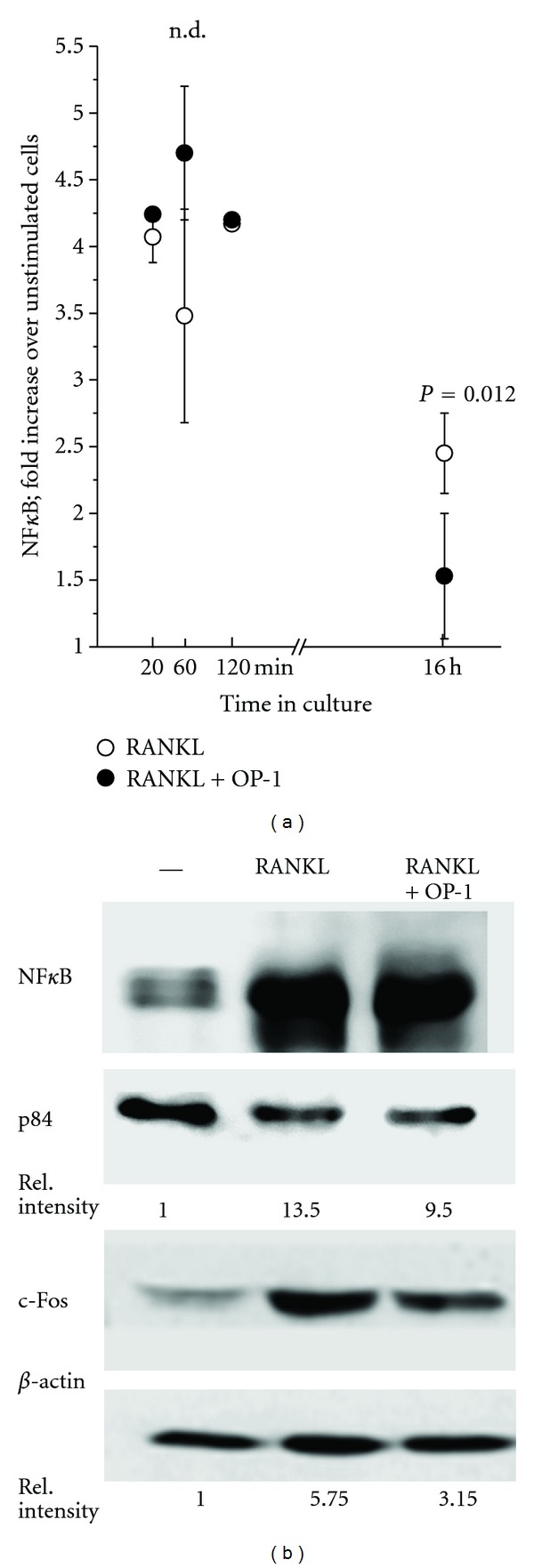
Translocation of NF*κ*B into the nucleus and induction of c-Fos: (a) NF*κ*B was determined by ELISA in the nucleus of monocytes treated with RANKL (∘) or RANKL + OP-1 (●) for the times indicated. The data points are compiled from experiments with cells of four individual donors (with exception of time point 120 min; here data of only one donor were available). By 16 h, the mean values obtained for RANKL and the RANKL + OP-1 treated cells were different as calculated by *t*-test. (b) By Western blotting, NF*κ*B was assessed in the nucleus in unstimulated cells, cells stimulated with RANKL or with RANKL + OP-1 (24 h). The relative intensity of the bands was determined using p84 as a nuclear housekeeping protein. c-Fos was determined in the cell lysates of monocytes stimulated as described above. Here actin was used as loading control.

Following activation by RANKL, NF*κ*B translocated to the nucleus. Under our experimental conditions, a 4-fold increase of NF*κ*B was seen within 20 to 120 min compared with unstimulated cells (Figure 3(a)). OP-1 by itself had no effect on the NF*κ*B translocation, nor did it modulate the RANKL-induced translocation. NF*κ*B, however, declined more rapidly in OP-1 treated cells. By 16 h, nuclear NF*κ*B was reduced by 64% (mean of experiments with cells of 4 individuals; the reduction was statistically significant as calculated by *t*-test (*P* = 0.0118) (Figure 3(a)). These data were confirmed by Western blotting (Figure 3(b); one of two experiments). c-Fos, which is downstream of NF*κ*B, was also upregulated following activation by RANKL within 24 to 72 h (data for 72 h are shown in Figure 3(b)). In the presence of OP-1 the abundance of c-Fos was reduced by 51.63% (±5.89%, mean ± SD of *n* = 3) (example in Figure 3(b)).

**Figure 4:**
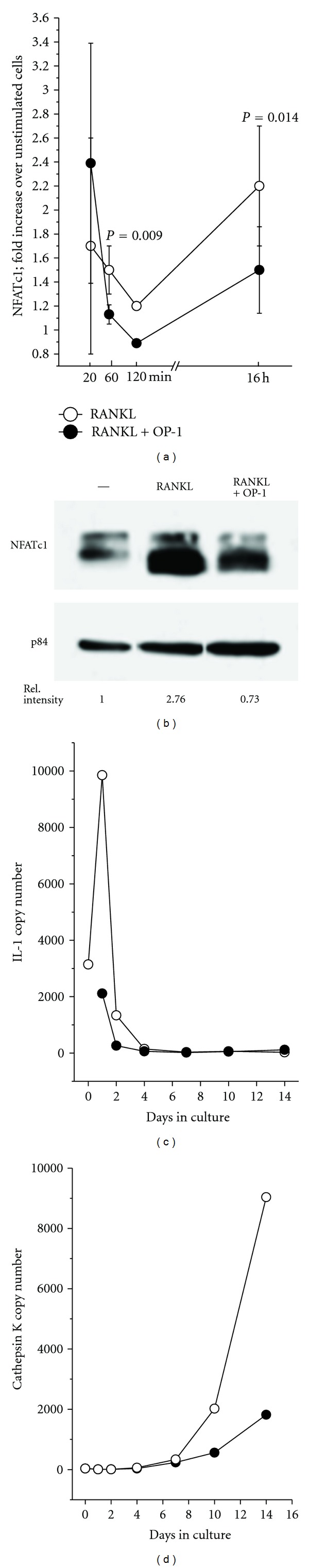
Effect of OP-1 on NFATc1: (a) NFATc1 in the nuclear fraction was determined by ELISA of monocytes treated with RANKL (∘), or RANKL + OP-1 (●) for the times indicated. Data derived from experiments with cells of four individual donors are shown (with exception of the time point 120 min), and differences of the mean values were calculated using *t*-test. (b) By Western blotting, the effect of OP-1 was confirmed (data for 24 h are shown). (c), (d) Various times after onset of culture, IL-1 and cathepsin K specific transcripts were determined by quantitative PCR (●); the open symbol (∘) shows the parallel experiment with addition of OP-1 (data are mean of duplicates; one of two experiments is shown).

RANKL also induced NFATc1 translocation into the nucleus within 5 to 20 min. Then NFatc1 declined, notably faster in the presence of OP-1. By 60 min, the difference between RANKL and RANKL-OP-1 treated cells was statistically significant (*P* < 0.01). Following prolonged culture, NFATc1 increased again, presumably due to an auto-amplification as it has been reported in the literature [32]. This increase was considerably reduced when OP-1 was present (data for 16 h are shown in Figure 4(a)) (2.23 ± 0.52 fold increase over unstimulated cells; mean of *n* = 4; in the presence of OP-1 only 1.52 ± 0.36 fold; the mean values are different with *P* = 0.014). We confirmed this finding by Western blotting: NFATc1 was seen for up to 72 h, and again a profound inhibition was seen, when OP-1 had been present during the culture (Figure 4(b); one of three experiments is shown). 

That BMP7/OP-1 inhibited NFATc1 was also shown in experiments using TNF*α* as stimulus: here, the increase in IL-1 message was inhibited by OP-1, as was the increase in cathepsin K, which is regulated in a NFATc1-dependent manner and is typically expressed by osteoclasts (Figures 4(c) and 4(d)).

### 3.3. Effect of BMP7/OP-1 on the Transcription Factor MafB

**Figure 5:**
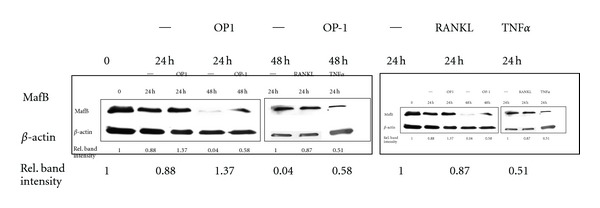
Effect of culture conditions and OP-1 on MafB expression: Monocytes were cultivated with or without OP-1 for the times indicated and MafB was determined by Western blotting. Actin was used as loading control. In another experiment, the effect of RANKL or TNF*α* on MafB expression was assessed (24 h).

MafB is strongly expressed by monocytes and monocyte precursors. Under our culture conditions, its expression decreased with time in culture, particularly when the monocytes were activated with RANKL or TNF*α* (examples in Figure 5). When OP-1 was present, MafB was conserved to some extent, by 48 h on average 48.5% (mean of three independent experiments).

## 4. Discussion 

Bone morphogenetic protein- (BMP-) 7/osteogenic protein- (OP-) 1 is a member of the transforming growth factor family and has numerous, highly diverse biological functions. With respect to bone turnover, BMP7/OP-1 is described as a promoter of bone formation, particularly by inducing cell proliferation and by recruiting stem cell to bone defects [24, 25]. We now described a novel function of BMP7/OP-1. In culture, BMP7/OP-1 inhibited the differentiation of monocytes to osteoclast. Inhibition was independent of the stimulus inducing osteoclastogenesis, and was also seen in osteoclast generation that occurred spontaneously (see Figure 2(b)). Using 1 *μ*g/mL OP-1 inhibited the osteoclast generation 50 to 60%: increasing the BMP7/OP-1 dose did not increase the inhibitory effect further. The inhibitory effect of BMP7/OP-1 that we found is in apparent contrast to previous data by others [33, 34]. In these studies a mouse-derived cell line was used (RAW), which shares some characteristics with mature monocytes, but differs in others, which might account for the different results. 

Because inhibition by BMP7/OP-1 occurred independently of the stimulus, we presumed that BMP7/OP-1 would interfere with downstream signalling events, and focussed on NF*κ*B, c-Fos, and NFATc1 because these were identified as crucial transcription factors for osteoclastogenesis [10–14, 35, 36]. NFATc1 is seen as the master switch, because it translocates to the nucleus in response to RANKL (and most likely to other stimuli), and persists for an extended period of time. It is continually synthesised due to an autoamplification mechanism, and controls transcription of genes required for osteoclastogenesis [11–13, 32]. Under our experimental conditions, BMP7/OP-1 interfered with this signalling cascade. Apparently, the initial nuclear translocation of NF*κ*B or NFATc1 was not affected, the persistence of these transcription factors in the nucleus, however, was reduced. Because osteoclast generation depends on a continuous action of NFATc1—data on NF*κ*B are not that stringent—for the transcription of osteoclast-typical genes, we presume that BMP7/OP-1 by downmodulating NFATc1 or preventing its synthesis inhibits osteoclast generation. In support of this presumption, BMP7/OP-1 in culture reduced the transcripts for cathepsin K, which is NFATc1 controlled and expressed exclusively by osteoclasts. That exogenously added cytokines inhibited osteoclastogenesis by reducing NFATc1 and c-Fos had been shown before for IL-10, which inhibits differentiation of primary mouse cells or mouse cell lines to osteoclasts [37, 38]. 

NFATc1 controls not only genes required for osteoclastogenesis, but also the transcription of a repressor for MafB [15–18]. MafB is highly expressed in myeloid cells and is required to obtain and to maintain the monocyte/macrophage status. Its downmodulation is crucial for osteoclastogenesis, because MafB, in turn, can inhibit transcription of relevant genes [16, 17, 39]. In the presence of BMP7/OP-1 MafB was preserved to some extent, (about 50%), which could contribute to the BMP7/OP-1 dependent inhibition of osteoclastogenesis. In line with our data that BMP7/OP-1 acts on early steps of the differentiation process is the observation that osteoclast formation was only inhibited when BMP7/OP-1 was added within the first 24 h of stimulation, but not when added later on.

Under our experimental conditions, some monocytes also differentiated to osteoclasts without adding a stimulus. This phenomenon had been reported before by others, and was attributed to already committed precursor cells [40]. On the other hand, a low-level activation due to the isolation procedure, particularly the positive selection by anti-CD14 coated beads, cannot be ruled out, and would be in line with the decline of MafB that we saw in monocytes cultured for 24 to 48 h. Of note, the decline of MafB was less pronounced in the presence of BMP7/OP-1: here approximately 50% of the MafB was preserved, suggesting a direct effect of BMP7/OP-1 on MafB expression, and a further explanation, how BMP7/MafB could inhibit osteoclast generation.

BMP7/OP-1 applied locally is used for therapy in patients with large bone defects or delayed fracture healing [26–30]. Whether or not inhibition of osteoclast generation contributes to the beneficial effects of BMP7/OP-1 in these patients remains to be shown. Because in patients with delayed fracture healing TGF*β*, which controls osteoclast generation and eventually induces their apoptosis [41–43], is reduced [44], it is feasible that exogenously added BMP7/OP-1 might substitute for TGF*β* activity.

In conclusion, a novel function of BMP7/OP-1 was described: the inhibition of osteoclast formation from monocyte precursor cells *in vitro*. Inhibition of osteoclast generation by BMP7/OP-1 could provide a further means to control unwanted bone degradation.
